# The Pathology, Diagnosis and Treatment of Diseases of Women

**Published:** 1873-01

**Authors:** 


*The Pathology Diagnosis and Treatment of Diseases of Women, including Diagnosis of Pregnancy.* By GRAILEY HEWITT, M. D., London, F. R. C. P. Second American Edition, from the third London. Revised and enlarged, with one hundred and thirty-two illustrations. Lindsay & Blakiston, Philadelphia: 1872.

This work is already in the hands of the profession, and but little need be said of it. As a treaties upon uterine pathology, it certainly holds first rank, and is fully up to the present views of gynacologists, and mostly in perfect harmony with other authors.

The *mechanical treatment* advocated by the author, is not so generally accepted by the profession. Upon this subject the work is pre-eminently the champion of mechanics. The Ring Pessary and Uterine Sound are chief reliances in cases of flexion and version, and almost all conditions of Uterine disease are met by mechanical aids.

For those who desire full instruction and careful illustration in the department nothing can equal the work before us; the philosophy of mechaics, and the modes of application are fully presented.

Ring pessaries and indeed all pessaries are held by many practitioners at a very great discount from former estimates, stem pessaries at a still lower rate of approval. In a great many cases it seems much better to let the uterus tip or flex or even prolapse, than to resort to too many "traps" for correction, but it may be that a few cases will be benefitted by mechanical support. Aside from the authors views in this respect no fault can be found with his book, indeed this cannot be called a fault since it presents his side thoroughly and fully, but the pessary question has two sides. The other side is *not* in accordance with the views of the author.

